# Structures, Properties, and Bioengineering Applications of Alginates and Hyaluronic Acid

**DOI:** 10.3390/polym15092149

**Published:** 2023-04-30

**Authors:** Shuping Zhang, Jiayu Dong, Renxue Pan, Zhenyang Xu, Mengyuan Li, Rui Zang

**Affiliations:** School of Materials and Chemistry, University of Shanghai for Science and Technology, Shanghai 200093, China; 202242281@st.usst.edu.cn (J.D.);

**Keywords:** alginate, hyaluronic acid, tissue engineering, drug delivery

## Abstract

In recent years, polymeric materials have been used in a wide range of applications in a variety of fields. In particular, in the field of bioengineering, the use of natural biomaterials offers a possible new avenue for the development of products with better biocompatibility, biodegradability, and non-toxicity. This paper reviews the structural and physicochemical properties of alginate and hyaluronic acid, as well as the applications of the modified cross-linked derivatives in tissue engineering and drug delivery. This paper summarizes the application of alginate and hyaluronic acid in bone tissue engineering, wound dressings, and drug carriers. We provide some ideas on how to replace or combine alginate-based composites with hyaluronic-acid-based composites in tissue engineering and drug delivery to achieve better eco-economic value.

## 1. Introduction

As the demand for novel materials in the field of bioengineering continues to grow, the pursuit of advanced functional materials has become a hotbed of research [1]. Numerous polymer-based material systems, such as microgels [2,3], liposomes [4,5], dendritic polymers [6], and micelles [7,8], have been developed. Given their application in living systems, these materials must degrade in a manner consistent with their intended function, possess mechanical properties suitable for their intended use, produce non-toxic biodegradation products, be readily absorbed or excreted [9], and not cause prolonged inflammation [10].

Polysaccharide-based biomaterials have garnered significant attention and research, owing to their excellent biocompatibility [11], biodegradability, low toxicity, and renewable prospects. Hyaluronic acid (HA), an acidic mucopolysaccharide with potent antioxidant, gelling, anti-inflammatory, and wound-healing properties, is ideally suited for use in the pharmaceutical and personal care industries [12]. For example, Xia [13] cross-linked HA with CMSS via the Schiff base reaction to form a biocompatible hydrogel adhesive for hemostasis. Additionally, it is used as a drug delivery agent and can be administered by different routes, such as nasal, oral, pulmonary, and gastrointestinal [14]. It has been reported that insulin-loaded HA nano-delivery systems exhibit an effective hypoglycemic effect [15]. Alginate, a natural polysaccharide extracted from seaweed, finds widespread use in the fields of tissue engineering, wound healing, and regeneration, primarily in the form of hydrogels [16]. For example, Yao [17] found that gelatin and sodium alginate composite hydrogels performed well in wound healing through in vitro models of secretion system experiments. Alginate for scaffold fabrication and stem cell regeneration has been a hot research topic in recent years [18]. A composite of glass nanoparticles and alginate has been shown to be useful for bone regeneration [19].

This paper undertakes a comprehensive review of the structure and properties of alginate and hyaluronic acid, and compares the differences in the application of their modified cross-linked derivatives in bone tissue engineering, wound dressings, and drug carriers.

## 2. Molecular Structure and Properties of Alginate and Hyaluronic Acid

### 2.1. Alginate

Alginic acid is a natural polysaccharide polymer compound rich in carboxyl groups (COOH^−^) and has been widely used in cell engineering, 3D bioprinting, drug delivery systems, medical dressings, and other fields [20]. The discovery of alginic acid can be traced back to 1881, when Stanford extracted alginic acid with sodium carbonate and precipitated it in an acidic solution, discovering D-mannuronic acid as one of the components of alginic acid. In 1950, Fischer and Dörfel used chromatographic techniques to identify L-guluronic acid in alginic acid. Brown algae such as kelp, macroalgae, Golden algae phylum, and sargassum, and bacteria such as nitrogen-fixing bacteria and Pseudomonas are the main sources of alginic acid [21,22].

**Figure 1:**
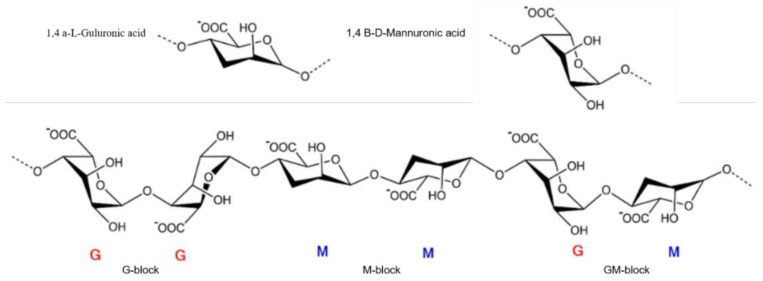
The conformation of monomers and blocks distribution of alginate salt. Reproduced with permission [39], copyright © 2023, American Chemical Society.

It is worthy to note that the extracts derived from seaweed are typically alginates, which are linear anionic block copolymers consisting of (M-block) β-D-mannuronic acid and (G-block) α-L-guluronic acid [23] (Figure 1). Alginate, with its plethora of oxygen-containing groups, including carboxyl and hydroxyl groups, is capable of forming intramolecular hydrogen bonds [24]. Certain alginate extracted from seaweed may also carry sulfate groups, while alginate-containing acetyl groups can be found in select bacteria.

The physicochemical properties of alginate depend on the M/G ratio [25], the block length, and the arrangement of repeating units in the biopolymer [26]. Therefore, the physicochemical properties of alginate can be influenced by where the extracted seaweed is grown and the season of harvesting. Alginate-based biomaterials have been developed to act in different forms in various fields. Among them, alginate hydrogels have been extensively studied because of their better hygroscopicity, biocompatibility, non-toxicity and flexibility, especially in tissue repair as well as in delivery systems. When high levels of G-blocks meet divalent cations, the alginate backbone forms a rigid hydrogel and the divalent cations bind to two oppositely placed G-blocks, forming an egg-box conformational arrangement. Alginate gels with more repeating G-block units are considered stiffer and more brittle [27]. In contrast, alginate gels characterized by a high proportion of M-blocks behave as soft and more elastic gels [28]. Alginate with a high content of M-blocks was reported to have reduced adhesion and showed immunostimulatory activity compared to alginate with a high content of G-blocks [29,30]. Additionally, the stirring speed [31], compression rate [32], and deformation percentage during the preparation process affect the mechanical strength of alginate gels.

Over the course of recent years, myriad in vivo and in vitro experiments have been conducted in order to assess the biocompatibility of alginate [33]. However, despite these endeavors, the effects of alginate on the human population remain a contentious and disputed issue. In a study conducted by Otterle [34], it was revealed that alginate with high M content engenders heightened immunogenicity; nonetheless, this claim has been challenged by other researchers, who have not encountered similar findings. It has been observed that immunogenic reactions that arise at the site of injection are frequently attributable to residual impurities, including, but not limited to, heavy metals and proteins. In addition, Lee [35] conducted a study in which commercially available, highly purified alginate gel was injected subcutaneously into mice, and it was determined that a significant inflammatory response was not detected. Thus, it can be deduced that alginate that has been purified several times does not elicit significant reactions when introduced into the human body [36].

Alginate can achieve structural and property changes such as mechanical strength, gelation properties and cell affinity by binding other biomaterials, immobilizing specific ligands (peptides and sugar molecules) and by physical or chemical cross-linking. Alginate is also one of the most important polymers used in the production of biomaterials (films, gels, hydrogels, nanofibers, gauze, etc.) that create and maintain a moist environment around wounds as a means of promoting rapid wound healing [37]. More importantly, alginate-based biomaterials have been used in drug delivery systems due to their cross-linking activity and gelling properties, and the role of alginate gels in wrapping islets for the treatment of type I diabetes has been demonstrated [38].

### 2.2. Hyaluronic Acid

**Figure 2:**
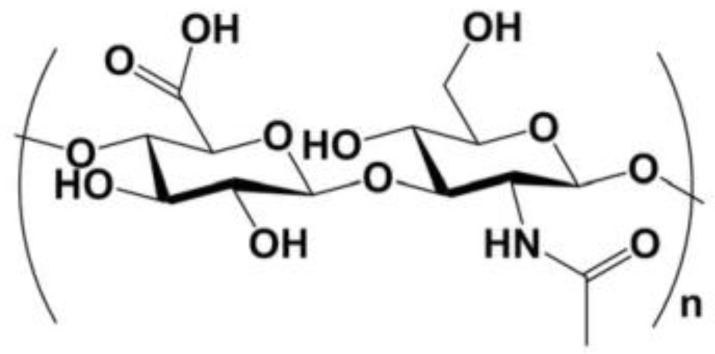
Molecular structure of hyaluronic acid. Reproduced with permission. Reproduced with permission [58]. © 2023 by the authors.

Hyaluronic acid (HA) is a glycosaminoglycan originally discovered in the vitreous humor of the bovine eye in 1934, followed by in vitro synthesis in 1964. Hyaluronic acid is made up of repeating D-glucuronic acid and N-acetyl-D-glucosamine linked by β1-3 and β1-4 bonds [40] (Figure 2) and polymerized into a macromolecule of over 30,000 repeating units. Hyaluronic acid is one of the largest components of the extracellular matrix (ECM) and is widely distributed in connective tissue, epithelial tissue, and neural tissue. According to the molecular weight distribution, HA can be classified as oligosaccharide (O-HA, <10 kDa), low-molecular-weight HA (LMW-HA, 10–250 kDa), medium-molecular-weight HA (MMW-HA, 250–1000 kDa), high-molecular-weight HA (HMW-HA, >1000 kDa), and ultra-high-molecular-weight HA (vHMW-HA, >6000 kDa). In the physiological environment, each carboxyl group in the HA molecule carries an anionic charge, and these charged carboxyl groups can establish hydrogen bonds with water molecules, thereby stabilizing the secondary structure of HA molecules. The formation of such hydrogen bonds is related to the molecular weight of HA, with higher molecular weights exhibiting higher stability, viscosity, and viscoelasticity [41,42].

The viscosity of HA is of paramount importance in the development of biochemical processes, tissue engineering, and drug delivery applications. The rheology of HA is influenced by several factors, including the ionic strength of the solution, pH, and temperature. When the pH of the solution is outside the range of 4 to 11, the HA molecule undergoes hydrolytic degradation, which can compromise the integrity of the polymer network and reduce its viscosity [43]. Interestingly, Kobayashi [44] discovered a remarkable linear relationship between the viscosity and molecular weight of HA. Changes in the molecular weight of HA, whether an increase or decrease by a factor of two, can lead to a ten-fold change in shear viscosity.

With the development of biomaterials, the biological properties of HA-based materials have received considerable attention. However, understanding the molecular weight of HA is still necessary for wound healing, scaffold development, and other cutting-edge technologies. The size of the molecular weight of HA also affects its biological effects and applications, to some extent [45] (Table 1). Additionally, the biocompatibility and cytotoxicity of HA are the most critical properties of HA as a biomaterial. Increasing the content of HA not only enhances the mechanical properties of HA–PL hydrogels, but also improves the cellular biocompatibility without exhibiting any cytotoxicity [46]. This finding is consistent with the research of Aunina [47].

Due to the large number of carboxyl and hydroxyl groups in the hyaluronic acid molecule, this allows hyaluronic acid to absorb exudates and enhance cell adhesion [55]. The free carboxyl and hydroxyl groups also allow HA to be richly chemically modified and many HA derivatives can be formed by chemical functionalization of carboxyl, hydroxyl and amide functional groups through methacrylate and carbodiimide reactions [56]. In addition, hyaluronic acid polymers can be completely hydrolyzed by the enzymatic action of hyaluronidase (HYAL), which allows hyaluronic acid composites to play an important role in tissue engineering and drug delivery systems [57].

### 2.3. Analytical Comparison of Alginate and Hyaluronic Acid

Although alginate and hyaluronic acid are both natural polymers that have shown potential for biomedical applications, they have different chemical structures, physical properties, and biological functions that make them suitable for different applications. The chemical structure of alginate is linear and it has relatively low viscosity and viscoelasticity [59]. Moreover, the hydrophobicity of alginate is high, which can interact with some proteins and, thus, affect its biological activity [60]. In contrast, the N-acetylglucosamine unit on the hyaluronan molecule causes the molecule to exhibit a branched structure [61], which gives hyaluronan a more spatial structure and allows it to form a more stable molecular conformation in aqueous solution. This leads to a higher viscosity and viscoelasticity of hyaluronic acid in an aqueous solution, resulting in better moisturizing properties.

In addition, alginate forms gels in the presence of divalent cations (e.g., calcium ions), which cross-link the polymer chains. The properties of the resulting hydrogel can be controlled by varying the concentration of alginate and the type and concentration of cross-linking agent. Hyaluronic acid, on the other hand, forms gels in a different way, either by physical entanglement of polymer chains or by chemical cross-linking with cross-linking agents to form hydrogels [62].

Alginate is biocompatible, biodegradable, and non-toxic, which makes it an excellent candidate for use in biomedical applications [63]. It has been used in wound healing, drug delivery, and tissue engineering. Alginate also has immunomodulatory properties that can be useful in the treatment of inflammation and autoimmune diseases. Hyaluronic acid plays an important role in tissue hydration, lubrication, and cell proliferation. It is found in many tissues throughout the body, such as in the skin, cartilage, and synovial fluid. Hyaluronic acid has been extensively studied for its use in tissue engineering, wound healing, and drug delivery [64]. It is also used in cosmetic products for its hydrating and anti-aging properties. We analyze and compare their similarities and differences in three aspects: bone tissue engineering, wound dressings, and drug carriers.

## 3. Analytical Comparison of Alginate and Hyaluronic Acid for Tissue Engineering and Drug Delivery Applications

### 3.1. Composites Based on Alginate and Hyaluronic Acid

Although natural polymers such as alginate and hyaluronic acid have been used in biomedicine for thousands of years, they still have limitations as single materials. The high hydrophilicity of hyaluronic acid makes it difficult to directly combine it with hydrophobic drugs; alginate hydrogels exhibit poor mechanical properties and, due to their inertness, alginates do not provide binding sites for cells [65]. However, numerous studies on natural biopolymers in recent decades have found that composites based on alginate and hyaluronic acid could make them more attractive biomaterials. Sithole [66] forms polyelectrolyte complexes from sodium alginate and poly (ethyleneimine) through solution interactions and designs scaffolds with mechanical capabilities for bone tissue engineering applications. Drozdova [67] prepared a composite macroporous hydrogel with hyaluronic acid/chitosan (Hyal/Ch) to load hydroxyapatite nanoparticles (nHAp), which can promote cell growth and proliferation and is expected to be used for bone tissue repair.

In addition, the morphology of alginate- and hyaluronic-acid-based composites is diverse. Thin films, sponges, aerogels, and microspheres have all been widely used in biomedical applications. We summarize some common composites in Table 2.

### 3.2. Bone and Cartilage Tissue Engineering

It is well-known that bone tissue has a natural ability to regenerate, but when the damage exceeds 2 cm, the bone injury will not be able to heal on its own [83]. With the advent of different biomaterials, bone and cartilage tissue engineering have received a lot of attention. The main goal of bone tissue engineering is to prepare a material that can be used for cellular remodeling by the body itself after artificial introduction of a bone defect [84]. Despite considerable progress in the development of biomaterials for bone tissue engineering applications, there are still some barriers to clinical translation. For example, while mechanical stability of scaffolds is required in many applications, rapid degradability is also required to accelerate tissue inward growth. Thus, composite materials offer the potential to develop tunable systems that meet a variety of requirements.

Scaffolds are temporary support structures for growing cells and tissues and play a vital role in supporting cells [85]. After a scaffold is implanted in the body, it can effectively support and act as a platform for host cell adhesion, proliferation, differentiation, and the formation of new bone tissue in vivo. The scaffolds should have several essential characteristics: (a) the surface can be adhered to by cells, promote cell growth, and retain the differentiation function of the cells [86]; (b) the scaffolds and their degradation by-products should not cause inflammation or be toxic in vivo [87]; (c) the implanted scaffolds must have adequate mechanical integrity [88]; (d) the porosity should be high enough to provide sufficient space for cell adhesion and extracellular matrix (ECM) regeneration during the culture process [89,90].

The HA scaffold material is prepared by different chemical modifications [91]. The derivatives obtained by chemical modification have different physicochemical properties from the natural polymers, but most of them can still maintain the biocompatibility and biodegradability of HA [92]. HA has many advantages as a tissue scaffold: (a) HA plays an important role in cell differentiation and cell growth [93]; (b) as HA can be involved in every step of the wound healing process, exogenous HA has the potential to provide a faster healing effect [94].

It has been shown that HA polymer scaffolds can be used in cartilage regeneration [95]. Several authors have used freeze-dried HA/chitosan [96] and HA/collagen scaffolds [97] to wrap auricular chondrocytes for cellular cartilage regeneration. Liu [98] implanted aortic endothelial cells into a scaffold formed by cross-linking thioglycolic acid (HA) with polyethylene glycol diacrylate (PEGDA) and transplanted it into a patellar groove defect in rabbit femoral articular cartilage. After 12 weeks, the defect was completely repaired. Lee [99] reconstructed articular cartilage using a chitosan–HA scaffold and showed that chondrocytes within the scaffold re-differentiated into hyaline cartilage structures and had collagen II expression. The hyaline cartilage obtained was transplanted to cartilage defects in the patellar groove of the rabbit knee and assessed by immunohistology: both hyaline cartilage and cartilage defects were restored, in contrast to the fibrocartilage formed in the control group.

**Figure 3:**
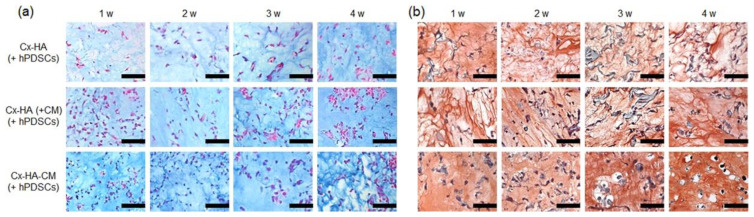
(**a**) Alcian blue (AB) and (**b**) SO-stained histological sections of implants of Cx-HA, Cx-HA (+CM), and Cx-HA-CM without (−) and with (+) hPLSCs. The implants were excised and analyzed after 1, 2, 3, or 4 weeks (magnification ×800; scale bars represent 25 μm). Reproduced with permission [100]. Copyright © 2023, the author(s).

Park [100] evaluated the utility of three novel hydrogels loaded with human periodontal ligament stem cells (hPLSC) for cartilage tissue engineering based on click-cross-linked hyaluronic acid hydrogels (cx-ha), namely, cx-ha, covalently linked and physically loaded cell regulatory protein-2 (CM), Cx-HA-CM, and Cx-HA(+CM). Cx-ha consists of the preparation of tetrazine-modified HA (HA-Tet) and transcyclooctene-modified HA (HA-TCO). In vivo cartilage differentiation results show that implantation of Cx-HA, Cx-HA(+CM), and Cx-HA-CM with hPLSC show a more organized tissue-like structure(Figure 3). The reason for this is that the action of CM on cell surface TGF-β receptors synergistically induces chondrogenic differentiation of hPLSC.

Scaffolds prepared from alginate-based composites have adjustable mechanical properties and low cost, and are considered to be one of the best materials for cartilage regeneration [101]. The use of regeneration promoters including bone marrow mesenchymal stem cells (MSCs) or supplementation with natural growth factors such as platelet-rich concentrates (PRC) has been shown to promote cartilage regeneration. The simultaneous use of MSCs and PRC may provide a synergistic effect and improve the repair of local cartilage damage, but this has not yet been demonstrated. Therefore, Samuel [102] conducted an experimental evaluation of whether the combined application of PRC could further enhance the repair capacity of alginate-coated MSCs in rabbit cartilage injury. Artificial total cartilage defects were modelled in the weight-bearing region of the medial femoral condyle of the bilateral knee joint in 30 New Zealand White rabbits. One month later, the 30 rabbits were randomly and equally divided into three treatment groups: alginate-coated PRC, MSCs, and PRC + MSCs composite grafts were implanted in the right knee joint. The results show that at 3 months, the grafts with PRC alone are as effective as MSCs in inducing cartilage defect repair. In contrast, the histological score is significantly higher in the PRC + MSC group (*p* < 0.05). At 6 months, in addition to the higher histological score and stronger staining, glycosaminoglycan per total protein content is significantly higher in the PRC + MSC group (3.4 ± 0.3 mg/mg) than in the MSC group (2.6 ± 0.2 mg/mg) or the PRC group (2.1 ± 0.2 mg/mg) (*p* < 0.05).

The application of alginate hydrogels has been reported to improve the histologic properties of tendon repair in vivo [103]. Yao [104] prepared a polycaprolactone (PCL)/sodium alginate (ALG) hydrogel composite scaffold loaded with melatonin MLT by electrostatic spinning technique, which had good mechanical properties and biocompatibility. The experimental results show that the PCL/MLT–ALG scaffold inhibits the production of reactive oxygen species (ROS), thus, promoting tendon repair. Here, we exemplify some examples of alginate- and hyaluronic-acid-based composites in bone tissue engineering in Table 3.

Alginate scaffolds facilitate cell regeneration and bone tissue formation, and can be used for the repair of some bone defects that require a relatively short time to form new bone tissue, such as bone severance and alveolar bone defects. The differences in the application of alginate and HA in bone tissue engineering have been investigated. Robert [112] used different concentrations of alginate and high-relative molecular mass HA hydrogels to culture osteoblasts MC3T3-E1 cells separately for a long period. The results show that the cells cultured with alginate show elevated bone sialic acid protein (BSP) mRNA levels and osteocalcin mRNA levels at later stages of maturation compared to control cells and cells cultured with high relative molecular mass HA, which are considered indicators of osteoblast differentiation and mineralization. This demonstrates that alginate hydrogels may be more suitable for bone tissue engineering applications than high-relative molecular mass hyaluronic acid hydrogels.

### 3.3. Wound Dressings

The skin, our body’s frontline defense against microbial invasion and dehydration, is crucial in maintaining our overall well-being [113]. However, trauma to the skin can lead to fluid loss and wound infection, potentially delaying or even preventing proper wound healing [114]. In order to avoid such deleterious outcomes, a variety of biomaterials, including sponges, films, and hydrogels, have been developed for tissue repair.

Hyaluronic acid (HA), the primary component of the skin’s extracellular matrix (ECM), plays a pivotal role in inflammatory responses, angiogenesis, and tissue regeneration. However, different molecular weights of HA have distinct effects on wound healing [115]. High-molecular-weight HA (HMW-HA) can suppress inflammation by regulating the recruitment of inflammatory cells, cytokine production, and stem cell migration. In contrast, low-molecular-weight HA (LMW-HA) promotes angiogenesis. Meanwhile, oligomeric HA (O-HA) stimulates endothelial cell migration and differentiation and fosters the proliferation of dermal fibroblasts and keratin-forming cells. As a result, composite materials based on hyaluronic acid have been developed for use in wound dressings.

In the realm of wound healing, the film serves as an essential mediator of water vapor transport, O_2_/CO_2_ exchange, and drug delivery. Wang’s latest creation [116], the montmorillonite/hyaluronic acid-gentamicin (MMT/HA–GS) multilayer film, is a prime example of such technology. This unique film, with its ability to respond to hyaluronidase (HAS) and bacterial infection microenvironments, facilitates the progressive release of GS, thus, accelerating wound healing. Not only does it offer the ability to load high doses of drugs (0.85 mg/cm^2^), but it also allows for intelligent drug release and peels away from the wound surface. In vitro and in vivo tests have shown that the film is highly bactericidal, and its mammalian cytological and histocompatibility properties are superior.

The sponge was able to absorb a large amount of wound exudate and maintain a moist environment at the wound site [117]. Rania [118] developed a chitosan–hyaluronic acid composite sponge enriched with andrographolide (AND). In vivo healing experiments in rats show that the hyaluronic acid composite sponge accelerates wound healing and reduces scar formation. Matsumoto [119] obtained cross-linked HMW-HA/LMW-HA sponges by immersing HMW-HA sponges in LMW-HA solution during experiments. Based on abdominal data from rats, the vascular area was higher in rats treated with HMW-HA/LMW-HA (≈0.05 mm^2^), which was in contrast to rats treated with HMW-HA sponges (≈0.03 mm^2^). This suggests that doping the sponges with LMW-HA accelerates the angiogenic process. Furthermore, the presence of myeloperoxidase (MPO) in neutrophils confirms that LMW-HA also inhibits the exuberant inflammatory response.

**Figure 4:**
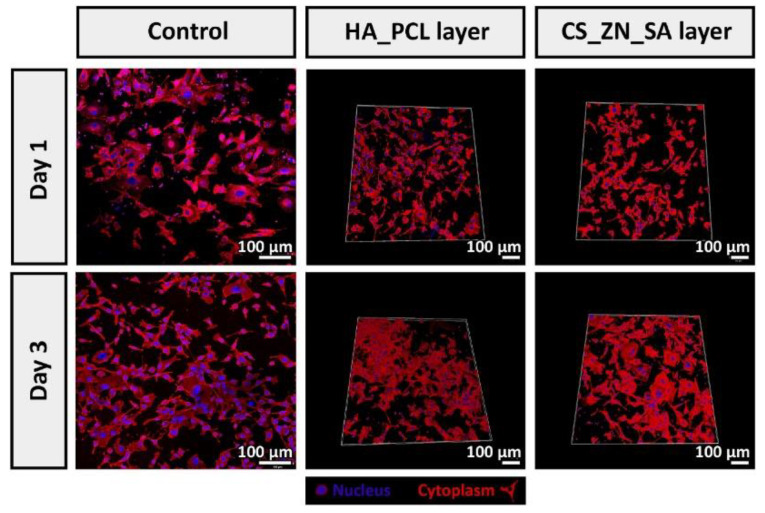
CLSM images of fibroblasts cultured at surface of culture plates (μ-slide 8 well Ibidi imaging plates (control)), HA_PCL and CS_ZN_SA membranes after 1 and 3 days. Blue channel: cell nuclei-labeled Hoechst33342; red channel: cytoplasm stained with WGA-Alexa 594 conjugate. Reproduced with permission [122] © 2023 Elsevier B.V. All rights reserved.

Nanofibrous membranes prepared by electrostatic spinning have a unique reticular structure [120] that promotes cell adhesion, growth, migration, and differentiation [121]. Figueira [122] used electrostatic spinning to prepare a bilayer electrostatic spinning membrane, with the upper layer consisting of hyaluronic acid and polycaprolactone, which acts as a physical barrier against external threats. The lower layer of the membrane consisted of chitosan and salicylic acid, which conferred anti-inflammatory and antibacterial activity to the lower layer. According to confocal laser scanning microscopy (CLSM images) (Figure 4), human fibroblasts maintain biological activity on the surface of the electrostatically spun membrane, and after 3 days, cell adhesion and proliferation occur. This suggests that this hyaluronic acid electrostatic-spun silk membrane has the potential to accelerate wound healing.

Wound healing is a multifaceted process that is influenced by various factors [18]. Among the many wound dressing options available, alginate-based dressings have demonstrated efficacy in creating an optimal wound-healing environment by removing wound exudate and providing a moist atmosphere [123].

Cheng [124] prepared a composite cellulose oxide nanocrystal (TOCN)/alginate film and sponge, which could offer superior wound healing properties. Through experimental evaluation, it was discovered that the water absorption capacity and chemical stability of the TOCN/SA composite sponges and films were higher than those of the single SA sponges and SA films. Their hemostatic properties were assessed by the bleeding volume and time to hemostasis in two injury models (rabbit liver trauma model and rabbit ear artery model). Astonishingly, the results indicate that the TOCN/SA composite sponge boasts an extraordinary hemostatic effect and completely degrades after a mere 3 weeks.

Shamshina [125] prepared a chitin–calcium alginate composite fiber dressing by dry–wet spinning. The ultimate stress value of the dressing was comparable to that of calcium alginate fiber and the water absorption capacity was consistent with that of currently marketed wound dressings. Wound-healing experiments show that rats achieve 95–99% wound closure on day 10 and complete closure on day 14. Tabassum [126] prepared wafers encapsulated with colloidal silver using sodium alginate and chitosan as raw materials, which were effective in reducing microbial infections in wounds using a freeze-drying method. Compared to commercially available products, the rate of wound healing was significantly increased in wounds treated with wafers containing colloidal silver. Here, we exemplify some examples of alginate- and hyaluronic-acid-based composites in wound dressings in Table 4.

In the realm of wound healing, both alginate- and hyaluronic-acid-based dressings are thought to be efficacious in speeding up the recovery process. Nonetheless, the question remains whether their coagulation effects on animals in vivo and in vitro warrant further exploration. At present, hyaluronic-acid-based wound dressings are more widely used in clinical applications, but their employment is subject to some limitations. For instance, hyaluronic acid can be degraded by hyaluronidase secreted by some bacteria found at the wound site, and under certain circumstances, it can even function as a ligand for microbial attachment [136]. When selecting the appropriate wound dressing, it is vital to consider the specific application scenario and the therapeutic effect that needs to be achieved [137].

Alginate-based wound dressings can be chosen when the desired dressing needs to have better mechanical properties and promote coagulation. Abou-Okeil [138] devised a cross-linked HA/sodium alginate (SA) film using calcium ions. In addition, he incorporated sulfadiazine (SD) with silver nanoparticles (Ag-NPs) alone or in combination as bioactive agents into this film. The findings demonstrate that the HA/SA/Ca2+/SD/Ag-NPs biofilm display the highest antibacterial activity, and it is proven to be the most effective for wound healing in rats. These outcomes provide some valuable insights into the preparation of wound dressings using alginate together with hyaluronic acid as a substrate.

### 3.4. Drug Delivery Systems

A comprehensive and technologically advanced system known as the drug delivery system (DDS) has emerged as an intricate solution for regulating the spatial, temporal, and dosing distribution of drugs within an organism. The DDS can be broadly categorized into two main types: conventional drug delivery systems (CDDS) and new drug delivery systems (NDDS) [139]. The CDDSs exhibit drug concentrations that are not constant during the treatment process, requiring frequent administration, which, in turn, leads to an abrupt surge in blood levels after each dose and exceeding toxicity limits [140]. In contrast, NDDS have gained significant traction and research attention in the pursuit of better therapeutic outcomes and mitigation of deleterious side effects on patients, owing to their ability to deliver drugs in optimal doses to specific regions of the body for gradual release, thereby minimizing the toxic ramifications of the drug [141].

In recent times, the use of HA and its derivatives in new drug delivery systems (NDDS) has garnered considerable attention, primarily as delivery vehicles for steroids, peptides, and various anti-cancer drugs [142]. These advanced drug carriers exhibit the remarkable ability to significantly enhance the residence time at the drug delivery site and reduce the number of doses, thereby circumventing the need for frequent administration [143].

Notably, Song [144] prepared a novel HA-modified graphene oxide (GO) nanohybrid for controlling the release of the anticancer drug Adriamycin (DOX) in tumor therapy. DOX achieved a loading efficiency of 42.9% on nanohybrids. In vitro release assays demonstrate that HA–GO–DOX nanohybrids could release DOX continuously and slowly in phosphate-buffered solutions. In vivo antitumor efficiency of HA–GO–DOX shows significantly enhanced tumor inhibition in H22 hepatoma-cell-bearing mice compared to free DOX and GO–DOX formulations.

The potential of hyaluronic acid hydrogels as drug carriers for treating inflammatory conditions has been extensively researched in recent times. Zhang [145] devises a carboxymethyl chitosan (CC) microsphere loaded with curcumin (CUR) and encapsulated it in an HA–gelatin (GE) composite hydrogel to treat inflammatory bowel disease (IBD). The formulation exhibits good sustained release performance with a drug release rate of 50% at 65 h, underscoring its potential as an ideal drug delivery vehicle. In vivo pharmacokinetic experiments show that the colonic tissue can sustain high levels of CUR for over 24 h, thereby providing valuable insights for developing novel oral delivery systems with controlled release behavior, particularly for treating IBD.

Acne has long been a scourge for millions of people worldwide, but a new route to treating this condition has been identified by Tolentino [146]. In this novel approach, chitosan and hyaluronic acid nanoparticles encapsulated with clindamycin are delivered to the hair follicle sebaceous glands. In vitro skin penetration experiments reveal that hyaluronic acid nanoparticles encapsulated with clindamycin increase clindamycin content in the hair follicle sebaceous glands (up to 77% of encapsulation), which is superior to commercial preparations (25%) and chitosan nanoparticles (53%). Furthermore, subsequent studies establish that the release of clindamycin is almost 100% when the skin is in the sebaceous state, signifying the enormous potential of this technique for treating acne.

Alginate hydrogels can be used as drug carriers to deliver low-molecular-weight drugs and macromolecular polymers, including proteins [147]. Liu [148] synthesized a temperature-sensitive copolymer, alginate–isopropyl acrylamide (alginate–PNIPAAm). The copolymer can form self-assembled micelles when the temperature is raised above the critical micelle temperature. In vitro release experiments show that the anticancer drug Adriamycin (DOX) is continuously released as micelles in PBS-buffered solutions. Furthermore, in vitro cellular experiments show that the gradually released DOX micelles can significantly enhance the uptake of DOX by multidrug-resistant AT3B-1 cells, thereby suggesting that alginate–PNIPAAm injectable hydrogels may well represent a potential therapeutic approach to address the vexing challenge of multi-drug resistance in the context of cancer therapy.

Alginate-based composites can also be used to construct hollow microencapsulated drug carriers by the layer-by-layer technique [149]. Shen [150] prepared a polyelectrolyte microcapsule, a chitosan–alginate multilayer bovine serum albumin capsule. The researchers encapsulated DOX in bovine serum albumin (BSA) gel capsules and performed in vitro antitumor experiments with the human breast cancer Adriamycin-resistant cell line MCF-7/ADR. The results show that the DOX-loaded BSA gel capsules are more lethal to MCF-7/ADR cells than free DOX. Additionally, the experimental findings evince some evidence of reversal of drug resistance, which is a vexing challenge in the context of cancer therapy. A nude rat transplantation tumor model showed that the encapsulated DOX had a longer retention time at the tumor site and a greater antitumor capacity in rats. There are various forms of alginate- and hyaluronic-acid-based composites in drug delivery (Table 5). It is not limited to hydrogels and capsules.

**Figure 5:**
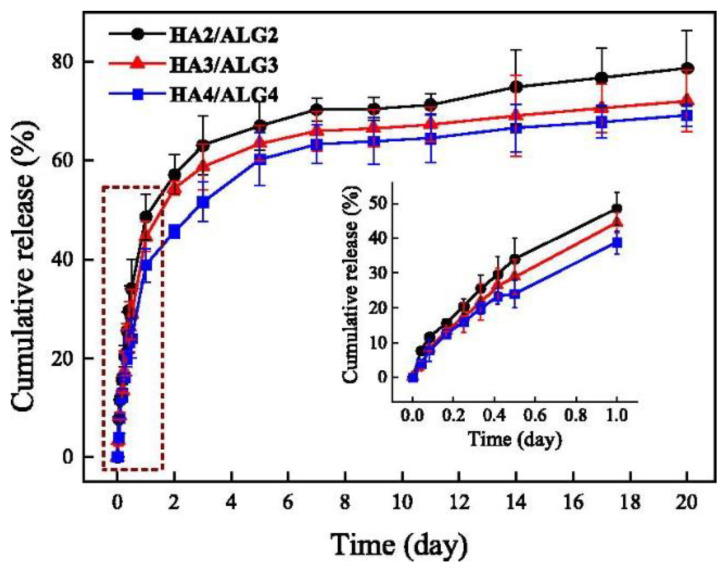
The BSA release profiles from drug-loaded HA/ALG hydrogels in PBS solution (pH 7.4) at 37  °C. Reproduced with permission [156]. Rights managed by Taylor & Francis.

In the realm of drug delivery systems, researchers have been investigating the potential of combining alginate and hyaluronic acid biocomposites. Zhang [156] devised a series of injectable hydrogels (HA/ALG) based on oxidized sodium alginate and acylated hyaluronic acid, utilizing bovine serum albumin (BSA) as a model for studying the controlled release properties of the drug. The results of in vitro release experiments reveal that the cumulative release of BSA from HA2/ALG2, HA3/ALG3, and HA4/ALG4 drug-loaded hydrogels after two days is approximately 72%, 69%, and 20%, respectively (Figure 5). Movahedi [157] encapsulated oxaliplatin (OXA) for the treatment of rectal cancer on folic acid (Folate)-coupled hyaluronic acid and encapsulated in alginate nanogels. The antitumor activity of free OXA, AL, HA/AL, HA/AL/OXA, and F/HA/AL/OXA nanogels were assessed in vitro. The results show that F/HA/AL/OXA nanogels show the highest antitumor activity in human colon cancer (HT29) cells, with a significant increase in apoptotic gene expression in HT29 cells compared to free OXA and empty nanogels. In conclusion, the combined utilization of alginate and hyaluronic acid in drug delivery systems has shown great promise, as evidenced by the results of these experiments. HA/ALG injectable hydrogels and F/HA/AL/OXA nanogels represent some exciting avenues for further exploration in this field. Here, we give some examples of alginate- and hyaluronic-acid-based composites in drug delivery, as shown in Table 6.

Alginate and hyaluronic acid have been identified as promising materials for the construction of DDSs. However, the application of alginate and hyaluronic acid in DDSs still faces some challenges. On the one hand, the molecular mass and structure of polysaccharides can be influenced by seasonal variations and environmental factors, which can affect the performance of the DDSs. On the other hand, the design of alginate- and hyaluronic-acid-based DDSs is still in its early stages, with many studies only providing brief evaluations of the properties and pharmacological effects of DDSs through in vitro and in vivo experiments. The interaction of DDSs with the human body, including the absorption, distribution, metabolism, and excretion of the carriers in the human body, requires further investigation. Nevertheless, researchers are hopeful that more alginate- and hyaluronic-acid-based nanomedicines will be approved for clinical applications. This would provide new and innovative solutions for drug delivery, potentially leading to improved therapeutic outcomes for patients.

## 4. Conclusions

Alginate and hyaluronic acid are emerging as a revolutionary class of biomaterials with unique biomedical applications. These versatile polymers have opened up new avenues for developing novel materials for cartilage regeneration, wound healing, and drug delivery. Tissue engineering has benefitted greatly from these polymers, as they can recreate the extracellular matrix (ECM) of the skin, thus, promoting stem cell differentiation or preserving the cellular phenotype. Current studies in this field are aimed at skin wound repair and repairing damaged cartilage, such as articular cartilage and ganglia. In the field of drug delivery, alginate- and hyaluronic-acid-based drug delivery systems (DDS) are gaining popularity as they enable slow, targeted release of drugs to specific cells. Research in this area is currently focused on developing therapies for inflammation and cancer treatment, such as containing or killing cancer cells. However, further research is needed to fully understand the properties and effectiveness of these biomaterials in various biomedical applications.

This comprehensive review article provides insight into the molecular structure and physicochemical properties of alginate and hyaluronic acid, as well as their versatile applications in emerging fields such as tissue engineering and drug delivery systems (DSS). In addition, we explore the tantalizing possibilities of replacing or even combining hyaluronic acid composites with alginate composites for greater biomedical efficacy. Given their rich and diverse origins, as well as their impressive biocompatibility, we are optimistic that more composites based on these two remarkable materials will be approved for clinical use, leading to superior therapeutic outcomes and considerable economic benefits.

## Figures and Tables

**Table 1:** Properties and applications of different molecular weights of hyaluronic acid.

Molecular Weight	Characteristic	Application	References
0.4–4 kDa	Non-apoptotic	Inducer of heat shock proteins	[48,49]
6–20 kDa	Immunostimulatory	Cell proliferation angiogenesis	[49,50]
200–250 kDa	Immunosuppressive	Wound dressing	[51,52]
<500 kDa	Anti-angiogenic activity	Space filler Natural immunologic depressant	[45,53,54]

**Table 2:** Composites based on alginate and hyaluronic acid.

Form	Composites	Technique	References
Nanofibers	Alginate/poly(ethylene oxide) Hyaluronic acid derivative	Electrostatic pinning Electrostatic pinning	[68] [69]
Microparticles	Calcium alginate/zein/hydroxypropyl Methylcellulose Hyaluronic acid/PLGA-/PLA	High voltage electrical discharge Double emulsion solvent evaporation	[70] [71]
Hydrogel film	Alginate/acacia Hyaluronic acid/Pt	Polymerization Cross-linking	[72] [73]
Hydrogels	Sodium alginate/acrylic acid Hyaluronic acid/ε-Polylysine	Electronic cross-linking and grafting Physical cross-linking	[74] [47]
Scaffolds	Alginate/gelatin/HEMA Methacrylated hyaluronic acid	Porogenation method and cross-linking Cross-linking	[75] [76]
Aerogels	Alginate/poly(vinyl alcohol) Hyaluronic acid/ε-Polylysine	Interpenetrating cross-linking Electrostatic interaction	[77] [78]
Film	Sodium alginate/antagonistic yeasts Hyaluronic acid/silk fibroin	Polymerization Cross-linking	[79] [80]
Sponge	Alginate/chitosan Hyaluronic acid/PAA/PVP	Cross-linking Solid/solution interface complexation	[81] [82]

PLGA: poly(lactide-co-glycolide); PLA: poly(lactide); HEMA: 2-hydroxyethyl methacrylate; PAA: poly(acrylic acid); PVP: polyvinylpyrrolidone.

**Table 3:** Alginate- and hyaluronic-acid-based composites in bone tissue engineering.

Material	Fabrication	Form	Main Outcomes	Reference
Collagen/oHAs/ hydroxyapatite	Lyophilization	Composite scaffold	Facilitate the osteogenic differentiation of MC3T3-E1 and BMSCs	[105]
HA/gelatin	Low-temperature polymerization	Cryogels	Restores and improves damaged tissue 3T3 cell adhesion is enhanced	[106]
HA/peptide	Electrostatic spinning	Electrospun fibrous scaffold	MC3T3-E1 has significant osteogenic differentiation and calcium mineralization.	[107]
HA/corn silk	Low-temperature polymerization	Antibacterial scaffold	Mesenchymal stem cells exhibit a high degree of bone differentiation	[108]
ALG/PGU/CNFs	Polymerization	Hydrogel nanocomposite	Improved success rate of bone regeneration	[109]
ALG/BCNs/CS	Layer-by-layer	Composite scaffold	Promoted the adhesion and spreading of MG70 cells and MC63T3-E3 cells	[110]
ALG/Hydroxyapatite	Electrostatic spinning	Nanocomposite fibers	More stable attachment of rat cranial osteocytes to the scaffold	[111]

CNFs: carbon nanofibers; PGU: polyglucuronic acid; BCNs: bacterial cellulose nanocrystals; CS: chitosan.

**Table 4:** Alginate- and hyaluronic-acid-based composites in wound dressings.

Type of Wound Dressing	Composite Materials	Applications	Reference
Sponges	HA, chitosan HA, chitosan, AgNPs	Wound healing Diabetic food ulcer	[118] [127]
Films	Alginate, lactobacillus plantarum HA, CSH, EEP	Burn wound Wound healing	[128] [129]
Hydrogels	Alginate, carboxymethyl chitosan HA-EDA, α-elastin	Chronic wounds Skin wounds	[130] [131]
Gauzes	Calcium alginate, chitin, gauze HA, silver sulfadiazine	Sinus surgery Ulcer healing	[132] [133]
Foams	Alginate, polyhexamethylene Biguanide, chitosan HA, ceramide	Wound dressing Atopic dermatitis	[134] [135]

CSH: cornstarch; EEP: ethanolic extract of propolis.

**Table 5:** Forms and techniques of alginate- and hyaluronic-acid-based composites in drug delivery.

Form	Composites	Technique	References
Tablets	Methylcellulose/hyaluronic acid/mannitol Alginate/diisopropylcarbodiimid	Direct compression Direct compression	[151] [152]
Capsules	HA/PAH/PLL Chitosan/alginate/BSA	Layer-by-layer Layer-by-layer	[153] [150]
Suppositories	HA/dehydroepiandrosterone sulphate Alginate–tamarind	Fusion molding Curing	[154] [155]

BSA: multilayer bovine serum albumin; PAH: poly(allylamine); PLL: poly(lysine).

**Table 6:** Alginate- and hyaluronic-acid-based composites for drug delivery.

Drugs	Polymer	Route	Formulation/Design Approach	References
Furosemide	Alginate/chitosan	Parenteral	Muco-penetrating nanoparticles for enhancement of oral bioavailability	[158]
Ciprofloxacin	Sodium alginate/ glycol/chitosan	Pulmonary	Grafted and spray-drying	[159]
Ciprofloxacin	Calcium alginate	Transdermal	Lyophilized hydrogels	[160]
Irinotecan	HA–NLC	Intravenous injection	Nanostructured lipid carriers	[161]
Doxorubicin	HA-PHis/TPGS2k	Transdermal	pH-sensitive mixed copolymer micelles	[162]
Paclitaxel	HA/DOCA	Transdermal	Redox-sensitive micelles	[163]
Paclitaxel	HA/solid lipid nanoparticles	Intravenous injection	Solid lipid nanoparticles	[164]

NLC: nanostructured lipid carrier; TPGS2k: d-α-tocopheryl polyethylene glycol 2000; HA-Phis: hyaluronic acid-g-poly(l-histidine); DOCA: deoxycholic acid.

## Data Availability

No new data were created or analyzed in this study.
